# Association between plasma level of superoxide dismutase and survival of patients with acute-on-chronic liver failure

**DOI:** 10.1186/s12876-022-02126-y

**Published:** 2022-02-05

**Authors:** Zhen Tian, Naijuan Yao, Yuchao Wu, Fei Wang, Yingren Zhao

**Affiliations:** 1 Department of Infectious Diseases, The First Affiliated Hospital of Xi’an Jiaotong University, 277 West Yanta Road, Xi’an City, Shaanxi Province China; 2 Department of Ultrasound, The First Affiliated Hospital of Xi’an Jiaotong University, 277 West Yanta Road, Xi’an City, Shaanxi Province China

**Keywords:** Oxidative stress, Inflammation, Acute on chronic liver failure, Prediction

## Abstract

**Background:**

Fewer than 50% of patients with acute-on-chronic liver failure (ACLF) recover spontaneously, and ACLF has high mortality without liver transplantation. Oxidative stress has been shown to mediate hepatic inflammation during acute liver failure (ALF). We wanted to see if a biomarker for oxidative stress might be used to measure the severity and prognosis of ACLF patients.

**Methods:**

A retrospective cohort of 124 ACLF patients, as well as healthy individuals, liver cirrhosis and ALF patients, was studied between January 2015 and September 2018. The levels of plasma superoxide dismutase (SOD) were detected using an ELISA commercial kit, and the Kaplan–Meier method was used for survival analysis.

**Results:**

Patients with ACLF had statistically higher plasma SOD levels than the controls did (healthy controls and liver cirrhosis patients); however, the levels did not differ from those in patients with ALF. The plasma SOD level may be an inexpensive, easily accessible, and significant independent prognostic index for mortality on multivariate analysis (HR = 1.201, 95% CI 1.001–1.403, *P* < 0.01) as well as the model for end-stage liver disease (MELD) score. A level of SOD > 428 U/mL was linked to a statistically significant increase in the likelihood of death or liver transplantation in ACLF patients. Combination of plasma SOD levels and MELD scores improved performance in measuring the severity and prognosis of ACLF patients.

**Conclusion:**

Patients with ACLF can be classified into high-risk and low-risk groups based on their plasma SOD levels at the time of admission to the hospital. The patient outcome is more closely connected with the combination of SOD level and MELD score than either value alone. This approach might be used to predict patient prognoses and prioritize liver transplant candidates.

## Background

Acute-on-chronic liver failure (ACLF) is defined as an acute deterioration of liver function in an individual with pre-existing chronic liver disease, resulting in multisystem organ failure and high short-time mortality [1]. Currently, there are few effective therapies apart from liver transplantation [2]. The etiologies of ACLF vary between territories, alcoholic liver disease (ALD) and nonalcoholic fatty liver disease (NAFLD) are common in American and European countries, while viral infections (especially HBV infection) play central roles in Asian countries. With a carrier rate of hepatitis B virus (HBV) surface antigen at approximately 8% in adults, chronic hepatitis B (CHB) infection is the major cause of ACLF in China [3].

Currently, oxidative stress is believed to play an important role in liver failure [4]. During ACLF, injured/dead hepatocytes significantly increase oxidative stress, which leads to additional hepatocyte loss and inhibits regeneration, resulting in a vicious cycle [5, 6]. Recent studies have shown that scoring systems for assessing severity and disease outcomes of ACLF have been developed [7]. All of these methods, however, focus on compromised liver functioning, but few research have focused on disease pathogenesis, particularly the increased systemic oxidative stress associated with ACLF. Since reactive oxygen species (ROS) play a role in ACLF, we wonder if a biomarker for oxidative stress may aid in forecasting the disease's severity, mortality, and prognosis.

Superoxide dismutase (SOD) reduces the harmful effects of ROS by converting poisonous superoxide to hydrogen peroxide. SOD staining in liver tissues revealed an increased expression of SOD2, also known as manganese-dependent SOD (MnSOD), in patients with acute liver failure (ALF), and the plasma SOD level was increased and associated with disease severity [8]. Since ALF has a high degree of systemic oxidative stress, the increased plasma SOD level might represent an adaptive response during disease development. However, none of the studies that the author is aware of have evaluated the severity and outcome of ACLF patients from the perspective of hepatic oxidative stress. The purpose of this research is to see if plasma SOD is a reliable predictor of ACLF patients.

## Methods

### Patients

A total of 124 ACLF patients were included in our research at the First Affiliated Hospital of Xi'an Jiaotong University in Shaanxi, China, from January 2015 to September 2018. This retrospective study was conducted in accordance with the Declaration of Helsinki. All participants provided written informed consent, and the study was approved by the Research Ethics Committee of the First Affiliated Hospital of Xi’an Jiaotong University, Shaanxi, China.

Blood samples were collected at admission and 14 days after hospital admission, and then stored at – 80 ℃ within 2 h of collection.

The Asian Pacific Association for the Study of the Liver (APASL) criteria were used to diagnose patients with ACLF: (1) a serum bilirubin ≥ 85 mol/L; (2) an international normalized ratio (INR) ≥ 1.5 or a prothrombin activity ≤ 40%; (3) any degree of encephalopathy and/or clinical ascites within 4 weeks; (4) and signs of persistent chronic liver disease [9].

The European Association for the Study of the Liver (EASL) criteria were used to diagnose patients with ALF: (1) coagulation abnormalities, typically with an INR ≥ 1.5 or a prothrombin activity ≤ 40%; (2) any degree of encephalopathy; (3) no preexisting cirrhosis, and with an illness duration of < 26 weeks [10].

Patients diagnosed with ACLF or ALF ranged in age from 18 to 75 years old.

**Fig. 1:**
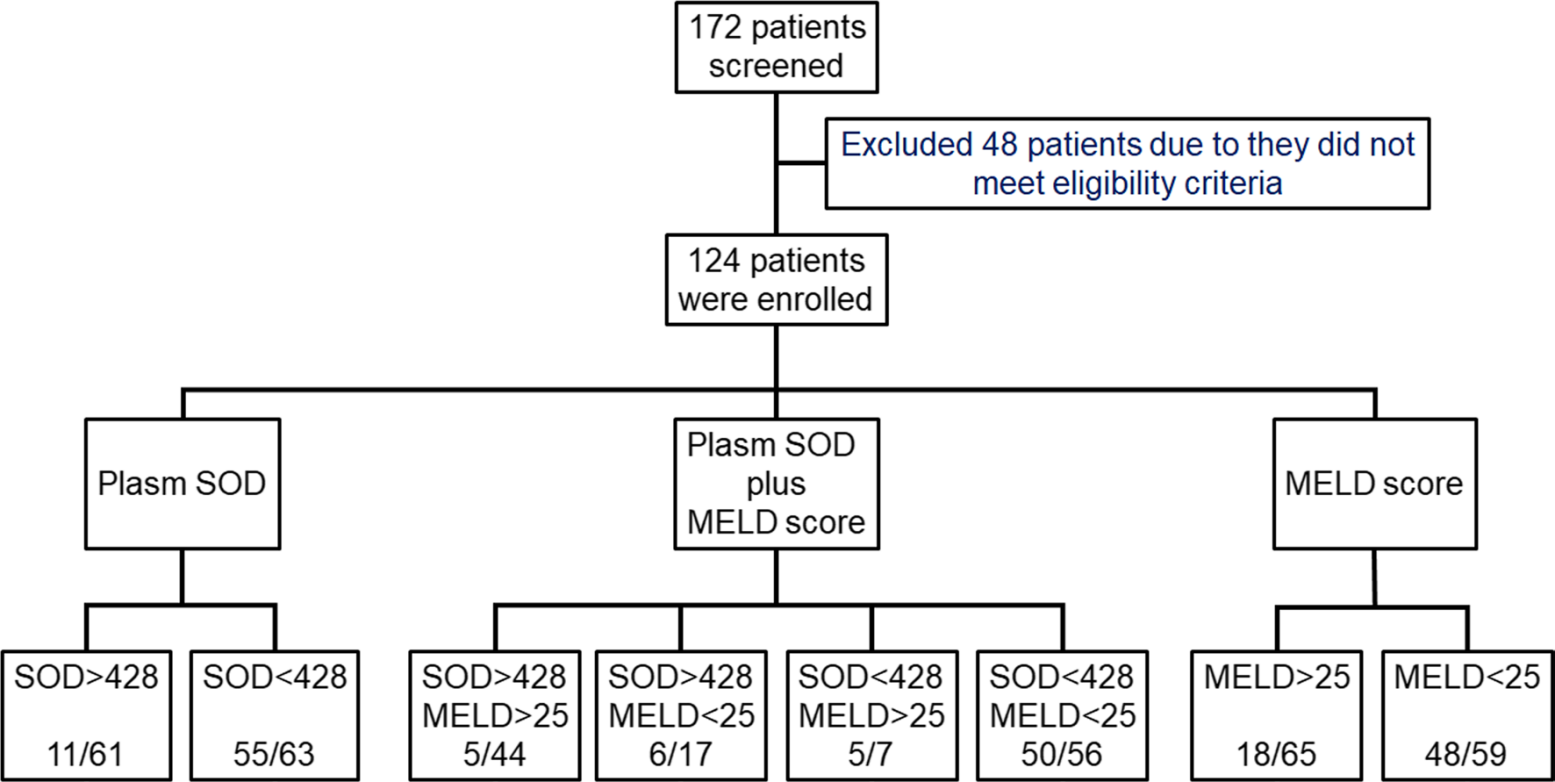
Patient disposition throughout the study

In our cohort, 48 patients were excluded for the following reasons: (1)manifestation of decompensated liver cirrhosis prior to ACLF diagnosis, such as ascites and variceal hemorrhage; (2) patients with portal hypertension who received a transjugular intrahepatic portosystemic shunt (TIPS); (3) patients pathologically diagnosed with or clinically suspected for having hepatocellular carcinoma (HCC); (4) other malignancies such as gastric cancer; (5) pregnancy; (6) HIV or hepatotropic virus infection other than HBV (Fig. 1).

During the same period, age- and sex-matched healthy participants and patients with liver cirrhosis were recruited as controls.

### Estimation of plasma SOD

According to the manufacturer's instructions, the blood SOD level was detected using a commercial ELISA kit (#EIASODC, Thermo Fisher Scientific, Waltham, MA, USA). The assay's sensitivity was 0.044 U/mL, and samples and standards were conducted in duplicate.

### Statistical methods

Means and standard deviations (SDs) are used to present the results. Demographics were compared using a chi-squared test or Fisher's exact test for categorical data, and a Wilcoxon rank sum test for continuous variables. ROC methodology was used to test the predictor values of SOD for disease severity and prognosis. The maximum of the sum of sensitivity and specificity was used to set cutoffs for continuous variables. Log-rank tests were used to compare Kaplan–Meier survival curves to 90 days after admission. Covariables with a *P*-value less than 0.05 were put into a forward multivariate analysis after univariate Cox regression analysis. SPSS version 16.0 was used to analyze the data (IBM Corporation, Somers, NY, USA) Differences were considered to be of statistical significance when the *P*-value < 0.05.

## Results

### Baseline characteristics

The present study cohort consisted of people who met the above-mentioned admission criteria for coagulopathy and encephalopathy. Table 1 shows the clinical baseline features of ACLF patients and controls in this investigation.

**Table 1:** Demographic data and clinical characteristics of controls and ACLF patients

Variables	Healthy control (n = 30)	Cirrhosis (n = 30)	ALF (n = 30)	ACLF (n = 124)
Age (yr)	42.54 ± 8.65	45.68 ± 5.39	45.23 ± 9.32	47.52 ± 11.88
Sex(M/F)	24/6	24/6	24/6	103/21
PT (%)	84.58 ± 15.77	76.36 ± 16.87	45.12 ± 11.27	37.76 ± 14.54*^#^
Fb (g/L)	2.68 ± 0.67	2.23 ± 0.95	1.65 ± 0.51	1.56 ± 0.71*^#^
INR	1.21 ± 0.19	1.37 ± 0.21	2.12 ± 0.34	2.09 ± 0.69*^#^
WBC(1 × 10^9^/L)	5.68 ± 1.54	4.85 ± 1.75	15.56 ± 6.45	14.46 ± 8.47*^#^
PLT (1 × 10^9^/L)	225.97 ± 38.54	115.04 ± 58.21	130.21 ± 21.35	99.90 ± 61.41*^#^
ALT (U/L)	25.21 ± 12.31	25.81 ± 12.32	355.75 ± 301.25	288.69 ± 396.73*^#^
GLU (mM)	4.39 ± 0.68	4.54 ± 0.98	5.23 ± 2.56	5.83 ± 3.32*^#^
TBIL (μM)	13.22 ± 3.79	19.88 ± 7.98	299.32 ± 178.23	320.62 ± 136.81*^#^
CHOL (mM)	3.97 ± 0.69	3.45 ± 0.91	2.78 ± 0.56	2.58 ± 0.90*^#^
CREA (μM)	49.67 ± 11.21	57.61 ± 9.81	65.23 ± 16.56	66.09 ± 22.22*^#^
MELD			25.05 ± 4.89	25.16 ± 4.33

ALT, alanine aminotransferase; CHOL, cholesterol; CREA, creatinine; Fb, fibrinogen; GLU, glucose; INR, international normalized ratio; PLT, platelet count; PT, prothrombin activity; TBIL, total bilirubin; WBC, white blood cell count

*Compared with healthy control *P* < 0.05

^#^Compared with cirrhosis *P* < 0.05

Only 124 ACLF patients out of 172 satisfied the criteria. Hepatitis B virus infection (70.16%) and alcohol usage (16.94%) were the most prevalent causes of underlying chronic liver illness; the most common causes of acute hepatic insult were hepatitis B virus reactivation (52.42%), alcoholic hepatitis (12.90%), and bacterial infection (27.42%).

The median age was 47.52 ± 11.88 years, with 83.06% of the population being male. Clinical events were defined by presence of complications. The most common consequence was HE (34.68%), while 24.19% of patients met the AKI criteria at the time of admission. Baseline clinical, laboratory parameters and in-hospital complications are summarized in Table 2.

**Table 2:** Baseline characteristics of ACLF patients

Variables	n = 124	Non-survivors n = 61	Survivors n = 63	*P* value
Age (yr)	47.52 ± 11.88	49.21 ± 1.37	45.89 ± 1.62	0.121
Sex(M/F)	103/21	51/10	52/11	0.874
ACLF etiology				
*Acute hepatic insult, n (%)*				
Alcoholic hepatitis	16 (12.90%)	8 (13.11%)	8 (12.70%)	0.888
HBV reactivation	65 (52.42%)	34 (55.74%)	31 (49.21%)	0.467
Bacterial infection	34 (27.42%)	17(27.87%)	17 (26.98%)	0.912
Others	9 (7.26%)	2 (3.28%)	7 (11.11%)	0.093
*Underlying CLD, n (%)*				
CHB	87 (70.16%)	45 (73.77%)	43 (68.25%)	0.499
ALD	21 (16.94%)	8 (13.11%)	13 (20.63%)	0.264
NAFLD	16 (12.90%)	8 (13.11%)	7 (11.11%)	0.732
*Clinic events at presentation, n (%)*				
Ascites	124 (100%)	61 (100%)	63 (100%)	
Jaundice	124 (100%)	61 (100%)	63 (100%)	
AKI	30 (24.19%)	20 (32.79%)	9 (14.29%)	0.015
HE	43 (34.68%)	31 (50.82%)	12 (19.05%)	< 0.01
Acute variceal bleed	17 (13.71%)	9 (14.75%)	8 (12.70%)	0.739
SBP	9 (7.3%)	4 (6.56%)	5 (7.94%)	0.767
*Parameter*				
PT (%)	37.76 ± 14.54	36.69 ± 221	38.81 ± 1.44	0.421
Fb (g/L)	1.56 ± 0.71	1.59 ± 0.11	1.54 ± 0.07	0.678
INR	2.09 ± 0.69	2.37 ± 0.11	1.82 ± 0.05	< 0.01
WBC(1 × 10^9^/L)	14.46 ± 8.47	7.60 ± 0.57	6.11 ± 0.35	0.027
PLT (1 × 10^9^/L)	99.90 ± 61.41	89.85 ± 6.44	109.6 ± 8.82	0.074
ALT (U/L)	288.69 ± 396.73	230.5 ± 44.43	345.1 ± 55.16	0.109
GLU (mM)	5.83 ± 3.32	6.15 ± 0.49	5.54 ± 0.36	0.316
TBIL (μM)	320.62 ± 136.81	404.1 ± 16.06	239.8 ± 11.77	< 0.01
CHOL (mM)	2.58 ± 0.90	2.52 ± 0.11	2.65 ± 0.12	0.420
CREA (μM)	66.09 ± 22.22	67.06 ± 2.89	65.17 ± 2.80	0.639
*Organ failure*				
Kidney, n (%)	11 (8.87%)	6 (9.84%)	5 (7.94%)	0.710
Cerebral, n (%)	20 (16.13%)	15 (24.59%)	5 (7.94%)	0.012
Coagulation, n (%)	43 (34.68%)	33 (52.46%)	10 (15.87%)	< 0.01
Circulation, n (%)	15 (12.09%)	8 (13.11%)	7 (11.11%)	0.732
Lung, n (%)	34 (27.42%)	22 (36.07%)	12 (19.05%)	0.034
*Prognostic score*				
MELD	25.16 ± 4.33	27.57 ± 0.51	22.83 ± 0.42	< 0.01

### ACLF was associated with high plasma SOD levels

**Fig. 2:**
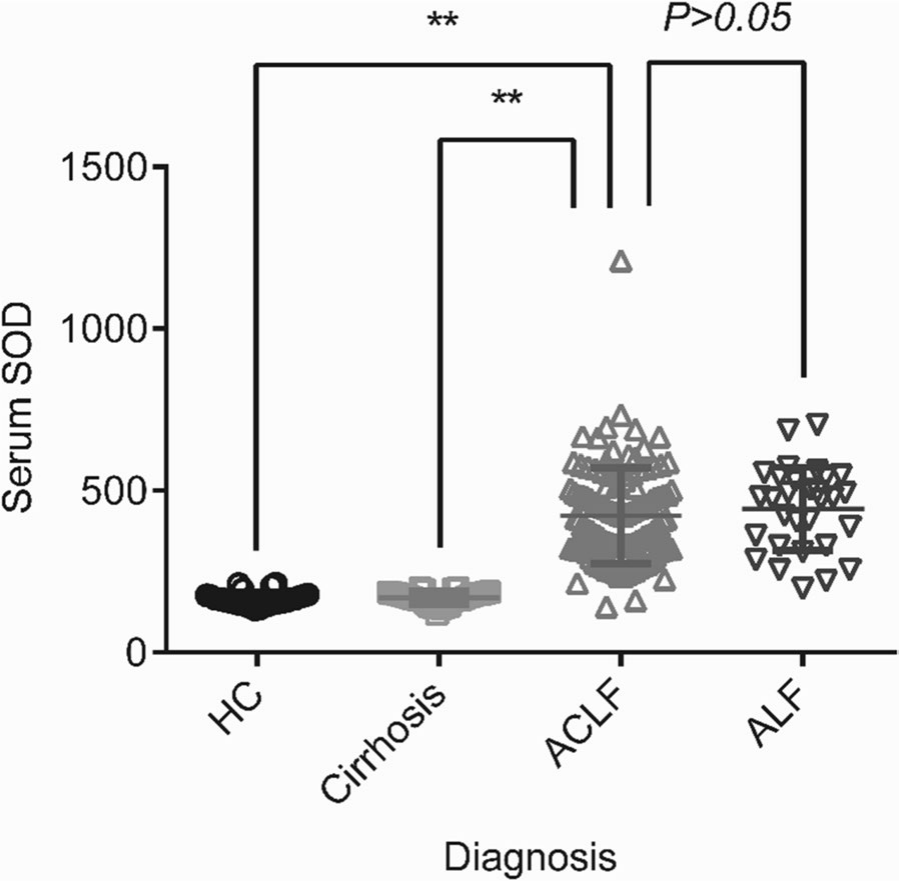
Values of the admission plasma SOD level (U/mL) in patients with cirrhosis, ACLF and ALF, **P < 0.01

Compared to healthy individuals, patients with ACLF had statistically higher levels of plasma SOD (423.1 ± 13.75 U/mL vs 164.2 ± 3.82 U/mL, *P* < 0.01), however, no significant difference was found in ACLF patients compared to patients with ALF (423.1 ± 13.75 U/mL vs 444.4 ± 23.58 U/mL,  *P* = 0.47) (Fig. 2). We wondered whether this increased SOD level in ACLF patients was a cirrhotic response. The plasma SOD level in patients with liver cirrhosis was then tested, and no significant differences were found compared to healthy controls (169.3 ± 3.69 U/mL vs 164.2 ± 3.82 U/mL, *P* = 0.34). However, a significant increase in the SOD level was observed in patients with ACLF compared to patients with liver cirrhosis (423.1 ± 13.75 U/mL vs 169.3 ± 3.69 U/mL, *P* < 0.01) (Fig. 2).

### Plasma SOD level was an independent risk factor for mortality in patients with ACLF

Here, we identified the potential risk factors for mortality of ACLF patients using univariate and multivariate Cox regression analyses. Table 3 showed that INR (HR = 6.782, 95% CI 2.711–16.996, *P* < 0.01), MELD score (HR = 1.454, 95% CI 1.261–1.676, *P* < 0.01), and plasma SOD level (HR = 1.214, 95% CI 1.009–1.419, *P* < 0.01) were significantly associated with mortality in ACLF patients. We therefore performed a forward multivariate analysis. The results revealed that MELD score (HR = 1.234, 95% CI 1.101–1.384, *P* < 0.01), and plasma SOD level (HR = 1.201, 95% CI 1.001–1.403, *P* < 0.01) were independent risk factors for mortality in ACLF patients.

**Table 3:** Uni-and multivariate logistic analysis of prognosis factors associated with survival in patients with ACLF

	Univariate			Multivariate		
	HR	95% CI	*P*	HR	95% CI	*P*
Age (yr)	1.024	0.994–1.056	0.123			
Sex(M/F)	1.079	0.422–2.760	0.874			
PT (%)	0.990	0.996–1.015	0.421			
Fb (g/L)	1.113	0.675–1.835	0.676			
INR	6.782	2.711–16.996	< 0.01	0.692	0.406–1.180	0.176
WBC(1 × 10^9^/L)	0.997	0.988–1.006	0.520			
PLT (1 × 10^9^/L)	0.995	0.989–1.001	0.078			
ALT (U/L)	0.999	0.998–1.000	0.121			
GLU (mM)	1.057	0.948–1.180	0.317			
TBIL (μM)	1.013	1.088–1.018	0.132			
CHOL (mM)	0.848	0.569–1.264	0.418			
CREA (μM)	1.004	0.988–1.020	0.637			
MELD	1.454	1.261–1.676	< 0.01	1.234	1.101–1.384	< 0.01
Plasma SOD (U/mL)	1.214	1.009–1.419	< 0.01	1.201	1.000–1.403	< 0.01

### Plasma SOD levels and MELD scores were associated with mortality or liver transplantation in patients with ACLF

**Fig. 3:**
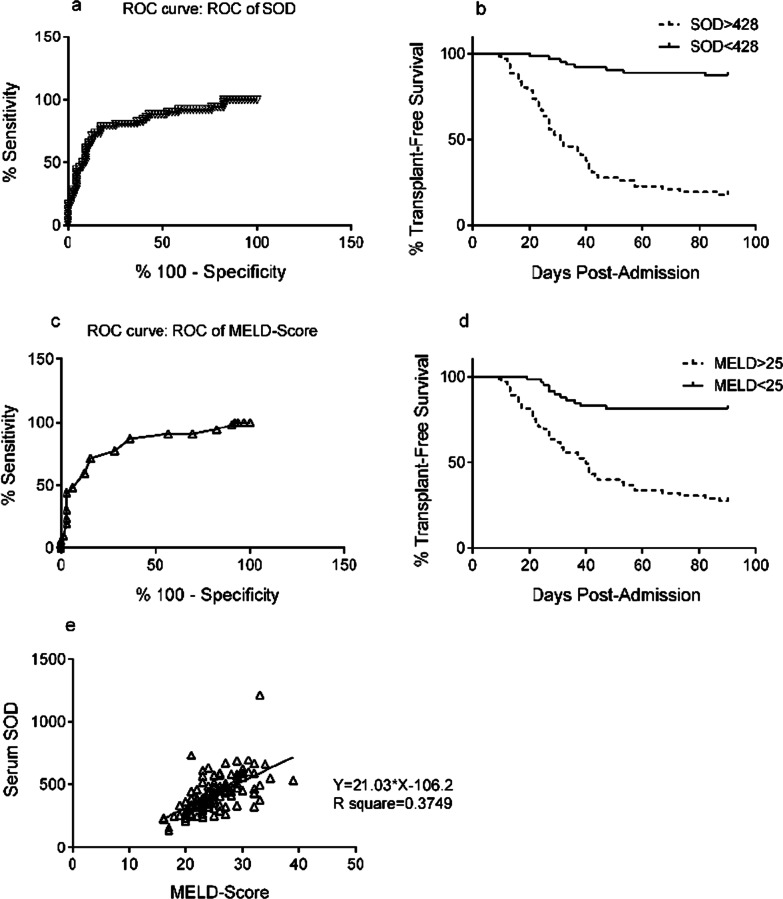
Kaplan–Meier analyses for survival according to the admission plasma SOD levels and MELD score. **a** ROC curve for plasma SOD. **b** Plasma SOD (above or below 428 U/mL) identifies ACLF patients with higher mortality. **c** ROC curve for the MELD score. **d** MELD score (above or below 25) identifies ACLF patients with higher mortality. **e** Plasma SOD was correlated with the MELD score

In the current cohort, 3 patients underwent liver transplantation, and there were 58 deaths without transplantation. Using the ROC methodology, the maximum sensitivity and specificity for the plasma SOD level as a predictor of death or transplantation within 90 days was 428 U/mL, and the corresponding value for the MELD score was 25 (Fig. 3a, c). Kaplan–Meier analysis showed that patients with a SOD value above this level had an increased risk of death or transplantation (*P* < 0.05) when censored at 90 days (Fig. 3b). Here, we assessed the predictive value of the MELD score in ACLF patients, and we found that patients with MELD score > 25 in the present study also had significantly greater mortality (Fig. 3d). Furthermore, a positive correlation was observed (Y = 21.03*X-106.2, R^2^ = 0.3749) when the SOD level was correlated with the MELD score (Fig. 3e).

These 124 ACLF patients were then divided into low SOD (SOD < 428 U/mL) and high SOD (SOD > 428 U/mL) according to plasma SOD level at hospital admission. Differences in the clinical and laboratory characteristics between the two groups of SOD are listed in Table 4. The MELD score in the high SOD group was higher than that in the low SOD group. Furthermore, patients with higher values of SOD had higher levels of PT, Fb, INR, WBC, ALT, TBIL, and mortality. The PLT, GLU, CHOL, CREA levels were not significantly different between the two groups.

**Table 4:** Clinical and laboratory characteristics among patients with different SOD values at hospital admission

	Low group	High group	*P*
	(SOD < 428, n = 64)	(SOD > 428, n = 60)	
Age (yr)	46.61 ± 1.62	48.50 ± 1.39	0.143
Sex(M/F)	50/17	44/13	0.739
PT (%)	37.48 ± 1.37	38.06 ± 2.29	< 0.01
Fb (g/L)	1.52 ± 0.07	1.62 ± 0.11	< 0.01
INR	1.85 ± 0.06	2.35 ± 0.10	< 0.01
WBC(1 × 10^9^/L)	6.06 ± 0.32	23.41 ± 15.78	< 0.01
PLT (1 × 10^9^/L)	100.60 ± 7.72	99.13 ± 8.02	0.894
ALT (U/L)	182.22 ± 25.35	388.51 ± 62.84	< 0.01
GLU (mM)	5.93 ± 0.41	5.67 ± 0.44	0.605
TBIL (μM)	213.12 ± 8.13	435.38 ± 12.19	< 0.01
CHOL (mM)	2.63 ± 0.11	2.53 ± 0.13	0.574
CREA (μM)	64.85 ± 2.82	67.43 ± 2.86	0.523
MELD	22.63 ± 0.42	27.87 ± 0.47	< 0.01
Plasma SOD (U/mL)	320.06 ± 7.87	545.78 ± 15.39	< 0.01

### Combination of the plasma SOD level and MELD score had an improved prognosis performance

**Fig. 4:**
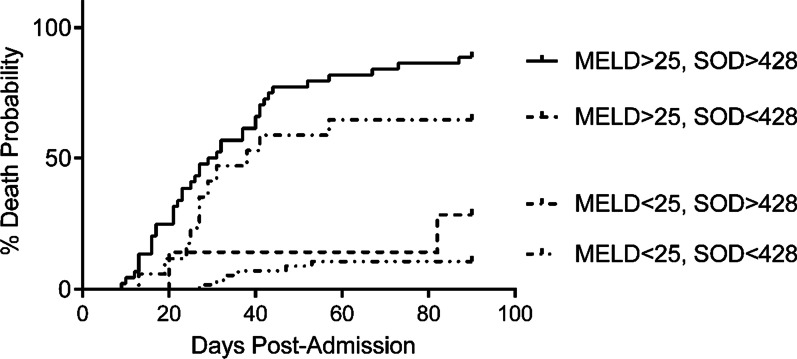
Assignment of ACLF patients into low-, intermediate-, or high-risk for 90-day mortality or transplant according to the combination of SOD and MELD

Performance characteristics were improved when the plasma SOD and MELD score were combined. A total of 89.29% of patients with MELD < 25 and SOD < 428 U/mL spontaneously recovered and survived at 90 days. Patients with SOD > 428 U/mL and MELD < 25 together with those whose SOD < 428 U/mL and MELD > 25 had a relatively diminished survival, patients exceeding both the SOD and MELD thresholds showed the poorest survival at 90 days (11.36%) (Fig. 4). Thus, the plasma SOD level is additionally informative in the prediction of transplantation or death when combined with the MELD score.

### Association of plasma SOD levels with oxidative stress

**Fig. 5:**
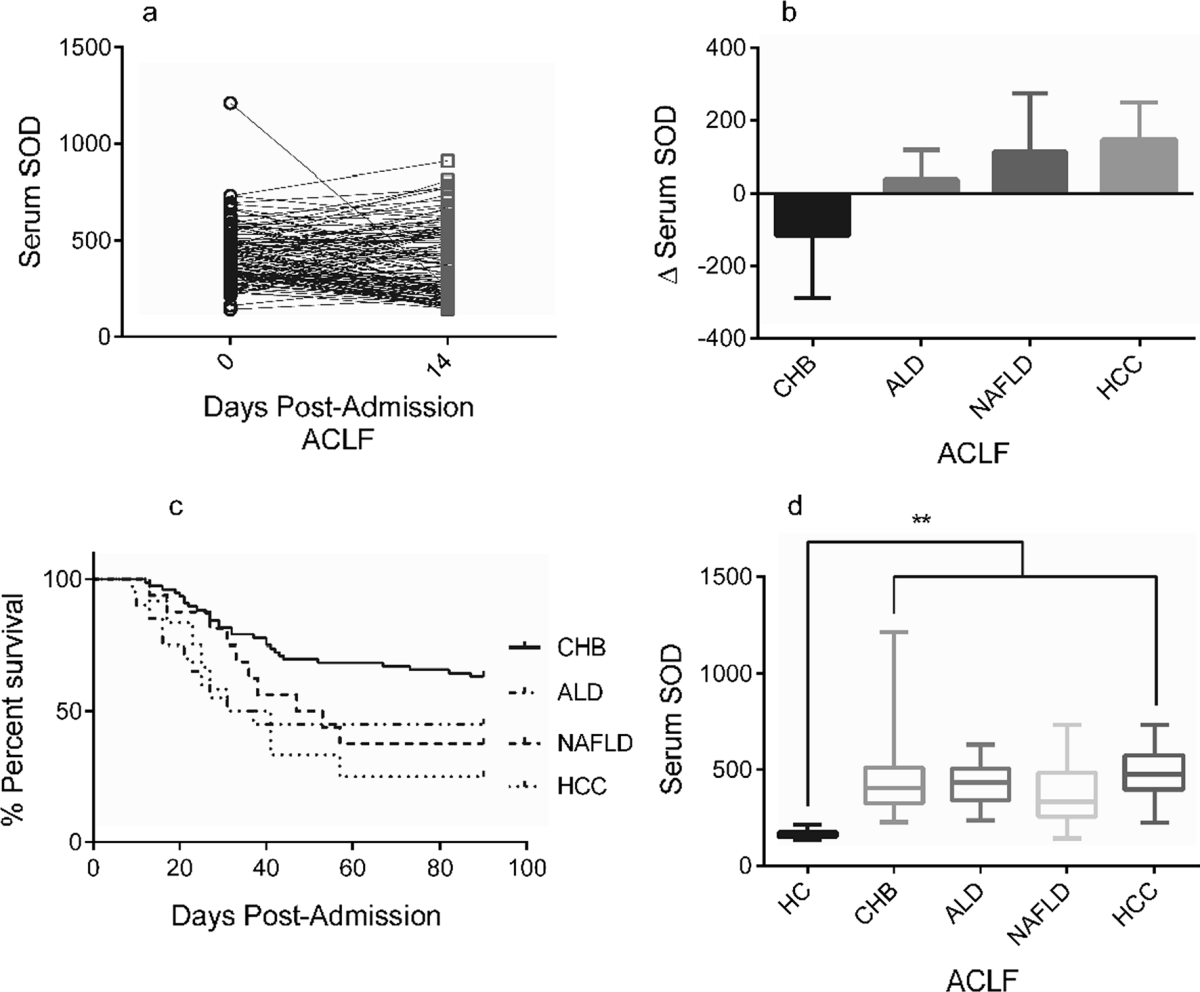
Plasma SOD levels in ACLF patients with different liver diseases. **a** Changes in the plasma SOD levels of ACLF patients. **b** Changes in the plasma SOD levels in ACLF patients based on different liver diseases. **c** Kaplan–Meier analyses for survival of ACLF patients based on different liver diseases. **d** Admission plasma SOD levels for ACLF patients based on different liver diseases, **P < 0.01

We previously found that the cytokine and chemokine levels vary during ALF, and we observed a significantly decreased SOD level during the remission stage compared to the progression stage in ALF patients [8]. However, the same result was not observed in these ACLF patients (Fig. 5a). In the present study we found that patients with ACLF based on ALD and NAFLD showed increased plasma SOD with disease progression (14 days after hospital admission), while ACLF patients due to CHB infection tend to recover with a decreased SOD level. We collected another 12 ACLF patients based on HCC and we found an increased plasma SOD level at 14 days (Fig. 5b). Kaplan–Meier analysis showed different risks of death or transplantation among these patients (Fig. 5c).

Recent studies have shown that ALD, NAFLD and HCC are disorders with increased systemic oxidative stress. In the current cohort, 21 ACLF patients had ALD, 16 had NAFLD, and another 12 ACLF patients had the basis of HCC. Although significantly higher than healthy controls, no significant differences were found in plasma SOD levels among patients with ACLF caused by CHB, ALD, NAFLD and HCC (431.4 ± 16.91 U/mL vs 430.7 ± 23.24 U/m vs 371.9 ± 44.91 U/mL vs 479.5 ± 39.76 U/mL) at the time of hospital admission (Fig. 5d).

## Discussion

ACLF is characterized by massive cell death and a quick decrease of liver function, which results in a high rate of short-term mortality. An acute insult based on chronic liver diseases leads to rapid and progressive liver failure and results in high mortality of approximately 50–90% at 90 days [11]. Here, we found a 59.93% mortality rate at 90 days among all these ACLF patients. King’s College Criteria, SOFA score and MELD score, together with the platelet to white cell ratio, albumin-bilirubin score, and log_10_AFP have been found to be simple models for evaluating the severity and disease outcomes of patients with ACLF [7]. However, most of these prognostic markers focus on impaired liver function and have been characterized by high specificity but low sensitivity. We thus wondered whether a marker reflecting disease pathogenesis and evaluating the severity and prognosis of ACLF exists.

Many human disorders, including ACLF, are invariably accompanied with excessive inflammation, inflammasome activation serves as a double-edged sword, contributing to both beneficial antimicrobial responses and cell death when overly active [12]. Endotoxemia and increased lipopolysaccharide (LPS) levels are common in ACLF patients due to increased gut permeability [13]. The liver sinusoids are made up of HSCs, sinusoidal endothelial cells, and Kupffer cells. These cells facing the sinusoidal lumen and in direct contact with the portal circulation serve as the gate against inflammatory stimuli, and they produce inflammatory cytokines when stimulated by the gut microbiota and microbial byproducts like LPS in septic liver injury [14]. Inflammatory cytokines are relayed to the parenchyma, causing hepatocyte destruction. Damaged/dead hepatocytes then dramatically enhance oxidative stress during ACLF. ROS are required for NLRP3 inflammasome activation, according to previous research [15]. Increased oxidative stress activates the inflammasome, which leads to further hepatocyte death and impedes regeneration, creating a vicious cycle.

In the present study, we, for the first time, assessed the prognostic value of the plasma SOD level, which increases as an adaptive response to elevated systemic oxidative stress during ACLF. We found that the circulating SOD level is markedly elevated in ACLF patients compared to the healthy control or liver cirrhosis groups, regardless of whether it is caused by CHB, ALD, or NALFD. The plasma SOD level has also been found to be associated with disease severity, as ACLF patients with SOD > 428 U/mL have significantly increased mortality.

This present study suggested that the plasma SOD level could serve as an independent predictor of mortality in ACLF patients. The MELD score has been widely used as a predictive criterion for assessing the severity of ACLF, but it is limited by the inner-laboratory variability in 3 components, creatinine and bilirubin related to the renal and hepatic function, together with INR [7], which can be affected by the usage of coagulation products. However, the plasm SOD level can be easily tested by ELISA. In addition, this study assessed the value of very early testing of the plasma SOD level as a predictor of the ACLF outcome.

This present study reported that the combination of plasma SOD level and MELD score had a higher predictive power than either the plasma SOD level or MELD score alone. Clinically, the most applicable use of this information is for predicting which patients will not die from ACLF without liver transplantation. Patients with ACLF who have SOD < 428 U/mL and MELD score < 25 are very likely to have spontaneous recovery and survive. In contrast, ACLF patients with SOD > 428 U/mL and MELD score > 25 have a survival rate less than 12% at 90 days. Thus, the addition of a simple, objective blood measurement, SOD, can be considered as a practical and significant adjunct to decision making in these extremely ill ACLF patients.

The present study showed that ACLF patients based on ALD, NAFLD, and HCC had higher plasma SOD levels than those based on CHB when censored at 14 days. ACLF is characterized by ROS production; ROS stimulates the NLRP3 inflammasomes, which regulate a variety of physiological responses and play a key role in liver failure. Mitochondria are the primary generator of ROS in hepatocytes that have been subjected to a "damage" injury (viruses, alcohol, environmental drugs, and therapeutic drugs) either immediately or chronically [16]. In NAFLD, mitochondria derived oxidative stress initiates a vicious cycle of exacerbated mitochondrial dysfunction and increased hepatocellular oxidative damage [17]; moreover, excessive production of ROS was found in HCC, which exceeds the capacity of the cells to move; therefore, it plays a crucial role in the occurrence and development of liver cancer [18]. Since oxidative stress plays a central role during ACLF, the increased oxidative stress during ACLF progression due to combination of ALD, NAFLD or HCC leads to much higher mortality rates compared to those in ACLF patients due to chronic HBV infection.

## Conclusion

The current study's findings were drawn from a large consortium over a 3-year period, demonstrating their generalizability; nonetheless, the current study's shortcomings include those inherent to retrospective research with possible biases, such as selection bias. In conclusion, the current investigation discovered an elevated level of SOD in ACLF patients, which was mostly due to increased oxidative stress during disease development. Plasma SOD testing at an early stage was found to be a good predictor of ACLF outcomes. Furthermore, developing strategies to reduce oxidative stress might give insight into pathogenic processes and could be a target for treating ACLF patients.

## Data Availability

The datasets used and/or analyzed during the current study are available from the corresponding author on reasonable request.

## Abbreviations

ACLF: Acute-on-chronic liver failure
ALD: Alcoholic liver disease
NAFLD: Nonalcoholic fatty liver disease
HBV: Hepatitis B virus
CHB: Chronic hepatitis B
MELD: Model for end-stage liver disease
ROS: Reactive oxygen species
SOD: Superoxide dismutase
MnSOD: Manganese-dependent SOD
TIPS: Transjugular intrahepatic portosystemic shunt
HCC: Hepatocellular carcinoma
SDs: Standard deviations
HSCs: Hepatic stellate cells

## References

[1] Bernal W, Jalan R, Quaglia A, Simpson K, Wendon J, Burroughs A (2015). Acute-on-chronic liver failure. Lancet.

[2] Blasco-Algora S, Masegosa-Ataz J, Gutiérrez-García ML, Alonso-López S, Fernández-Rodríguez CM (2015). Acute-on-chronic liver failure: pathogenesis, prognostic factors and management. World J Gastroenterol.

[3] Zhao RH, Shi Y, Zhao H, Wu W, Sheng JF (2018). Acute-on-chronic liver failure in chronic hepatitis B: an update. Expert Rev Gastroenterol Hepatol.

[4] Borrelli A, Bonelli P, Tuccillo FM (2018). Role of gut microbiota and oxidative stress in the progression of non-alcoholic fatty liver disease to hepatocarcinoma: current and innovative therapeutic approaches. Redox Biol.

[5] Xie F, Dong J, Zhu Y (2019). HIF1a inhibitor rescues acute-on-chronic liver failure. Ann Hepatol.

[6] Wang Y, Chen C, Qi J (2019). Altered PGE2-EP2 is associated with an excessive immune response in HBV-related acute-on-chronic liver failure. J Transl Med.

[7] Wu FL, Shi KQ, Chen YP, Braddock M, Zou H, Zheng MH (2014). Scoring systems predict the prognosis of acute-on-chronic hepatitis B liver failure: an evidence-based review. Expert Rev Gastroenterol Hepatol.

[8] Tian Z, Chen Y, Yao N (2018). Role of mitophagy regulation by ROS in hepatic stellate cells during acute liver failure. Am J Physiol Gastrointest Liver Physiol.

[9] Sarin SK, Kedarisetty CK, Abbas Z (2014). Acute-on-chronic liver failure: consensus recommendations of the Asian Pacific Association for the Study of the Liver (APASL) 2014. Hepatol Int.

[10] Wendon J, Cordoba J, Dhawan A (2017). EASL clinical practical guidelines on the management of acute (fulminant) liver failure. J Hepatol.

[11] Pamecha V, Kumar S, Bharathy KG (2015). Liver transplantation in acute on chronic liver failure: challenges and an algorithm for patient selection and management. Hepatol Int.

[12] Mehta G, Mookerjee RP, Sharma V, Jalan R (2015). Systemic inflammation is associated with increased intrahepatic resistance and mortality in alcohol-related acute-on-chronic liver failure. Liver Int.

[13] Jia Y, Ma L, Wang Y (2020). NLRP3 inflammasome and related cytokines reflect the immune status of patients with HBV-ACLF. Mol Immunol.

[14] Li J, Zhao YR, Tian Z (2019). Roles of hepatic stellate cells in acute liver failure: from the perspective of inflammation and fibrosis. World J Hepatol.

[15] Jiang C, Jiang L, Li Q (2018). Acrolein induces NLRP3 inflammasome-mediated pyroptosis and suppresses migration via ROS-dependent autophagy in vascular endothelial cells. Toxicology.

[16] Blajszczak C, Bonini MG (2017). Mitochondria targeting by environmental stressors: implications for redox cellular signaling. Toxicology.

[17] Kim SY, Jeong JM, Kim SJ (2017). Pro-inflammatory hepatic macrophages generate ROS through NADPH oxidase 2 via endocytosis of monomeric TLR4-MD2 complex. Nat Commun.

[18] Li Y, Zuo H, Wang H, Hu A (2019). Decrease of MLK4 prevents hepatocellular carcinoma (HCC) through reducing metastasis and inducing apoptosis regulated by ROS/MAPKs signaling. Biomed Pharmacother.

